# Brands with personalities – good for businesses, but bad for public health? A content analysis of how food and beverage brands personify themselves on Twitter

**DOI:** 10.1017/S1368980021001439

**Published:** 2022-01

**Authors:** Tenay Greene, Carla Seet, Andrea Rodríguez Barrio, Dana McIntyre, Bridget Kelly, Marie A Bragg

**Affiliations:** 1 Department of Population Health, NYU School of Medicine, 180 Madison Ave, 3^rd^ Floor, New York, NY 10016, USA; 2 Early Start, School of Health and Society, University of Wollongong, Northfields Avenue, Wollongong, NSW 2522, Australia; 3 Public Health Nutrition Program, School of Global Public Health, New York University, 665 Broadway, 11th Floor, New York, NY, USA

**Keywords:** Social media marketing, food marketing, branding, twitter

## Abstract

**Objective:**

To examine the extent to which food and beverage brands exhibit personalities on Twitter, quantify Twitter users’ engagement with posts displaying personality features and determine advertising spending across these brands on Twitter.

**Design:**

We identified 100 tweets from 10 food and beverage brands that displayed a ‘personality’, and 100 ‘control’ tweets (i.e. a post by that brand on the same day). Our codebook quantified the following personification strategies: (1) humour; (2) trendy language and (3) absence of food product mentions. We used media articles to quantify other personification strategies: (4) referencing trending topics; (5) referencing current events; (6) referencing internet memes and (7) targeting niche audiences. We calculated brands’ number of tweets, re-tweets, ‘likes’, and comments and report the relationship between advertising spending and retweets per follower.

**Setting:**

Twitter posts.

**Participants:**

Ten food and beverage brands that were described in media articles (e.g. Forbes) as having distinct personalities.

**Results:**

Personality tweets earned 123 013 retweets, 732 076 ‘likes’ and 14 806 comments, whereas control tweets earned 61 044 retweets, 256 105 ‘likes’ and 14 572 comments. The strategies used most included humour (*n* 81), trendy language (*n* 80) and trending topics (*n* 47). The three brands that spent the most on advertising had similar or fewer retweets per follower than the four that spent relatively little on advertising.

**Conclusions:**

Some food and beverage brands have distinct ‘personalities’ on Twitter that generate millions of ‘likes’ and retweets. Some retweets have an inverse relationship with advertising spending, suggesting ‘personalities’ may be a uniquely powerful advertising tool for targeting young adults.

Exposure to food advertising can contribute to poor diet^(1)^, and poor diet is a major risk factor for obesity, diabetes, CVD and some forms of cancer^(2,3)^. The majority of food advertising research has focused on television advertisements^(1,4)^, but food and beverage companies have begun to leverage the popularity of social media to engage with millions of consumers online^(5)^. Companies now spend $2·9 billion per year on advertising through Twitter alone^(6)^, and Coca-Cola allocates 20 % of their annual $4 billion marketing budget to social media^(7)^. One study that surveyed 1056 participants globally noted that 64 % of people surveyed who follow brands on social media were already fans of the brand or product before they decided to follow, suggesting companies face a unique challenge when trying to attract new followers^(8)^. Another survey showed that 37 % of over 200 000 respondents between the ages of 16 and 24 years said entertainment was the most important reason for using social media^(9)^.

Brands can attempt to attract followers by creating an entertaining human personality for their social media account. Brand personalities appeal to consumers by reflecting desirable lifestyles and attitudes, thereby producing a positive association with a brand’s products^(10–12)^. Some companies have aimed to engage with Twitter users using ‘the voice of the witty millennial’ to give the brand its own sarcastic, humourous or edgy personality through its Twitter account^(13)^, or use brand personalities to comment on current events, play into internet trends, and respond to rude or disrespectful comments in sarcastically humourous ways, also known as ‘clapbacks’ (see Table 1 for definitions of internet terminology used)^(13–17)^. For example, the US fast-food chain Denny’s started consistently using a ‘quirky-surreal-teen-blogger persona’ in 2013^(13)^ and increased its follower count by 132·8 % in 1 year^(15)^. In a recent descriptive study of how beauty brands employ Facebook to increase brand awareness and reinforce brand loyalty, findings revealed that most of the six beauty brands analysed posted content and activities that directly engaged consumers – via open-ended questions, Q&As or beauty polls – as opposed to other types of activities, such as simply promoting their products^(18)^.

**Table 1.** Important internet and Twitter-specific terms and definitions

Term	Definition
Tweet	The basic form of communication on the social media site Twitter. It is an original post made by a user in text, image, gif or video form.
(Twitter) Page	The location owned by a Twitter user where their original and shared tweets can be viewed by their followers.
(Twitter) Feed	The location viewed by a Twitter user where they can see the original and shared tweets of the users they follow.
Retweet	n. A Twitter-specific metric that signifies when a user has shared a tweet created by someone else on their own page to their own followers v. A Twitter-specific action that occurs when a user shares a tweet created by someone else on their own page to their own followers
‘Like’	n. A common social media metric across many platforms including Twitter that signifies when a user has reacted positively to a tweet created by someone else v. a common social media action that occurs when a user reacts positively to a tweet created by someone else
Comment	n. A common social media metric and/or artefact common across many platforms including Twitter that signifies when a user who views a tweet has responded to the tweet with their own text, image, gif or video v. A common social media action that occurs when a user responds to a tweet created by someone else with their own text, image, gif or video
Trending Topic	A topic that was mentioned and retweeted so often on Twitter that it has stood out as one of the ‘most’ discussed topic on Twitter that day OR any topic that was discussed as part of mainstream discussions by major news outlets.
Meme	An image, video, gif, text format, clause or word that is used in a specific way on the internet for humourous purposes, its meaning obvious to those ‘in-the-know’ but often unintelligible to those outside of that collective consciousness.
Clapback	A response to a hurtful or negative message that is meant to cut the opponent down in a way that may be humourous for its bluntness, accuracy and succinctness.

Brands may rely on Twitter to grow their social media fan base, given more than 152 million people worldwide use Twitter daily^(6)^. In the USA, more than 22 % of adults^(19)^ use Twitter. One study examined follower demographics from a sample of 26 food and beverage accounts on Twitter and showed that 77·7 % of the followers were from the USA and 65·8 % of the followers were aged 18–24 years^(20)^. In another study, young adults aged 18–34 years reported that their intention to follow brands on Twitter and Facebook was contingent on if they believed following those brands was an activity easy to accomplish; if following those brands benefited them in some way; and whether their friends or other users follow those brands^(21)^.

Social cognitive theory informs why young adults – the age group most likely to follow food and beverage brands on Twitter^(20)^ – may be particularly susceptible to brand personalities on social media^(22)^. First, the visibility of other users’ reactions to brand content through the number of likes, comments and retweets a tweet accumulates may influence whether or not an individual thinks that they should also respond to that tweet^(23–25)^. Second, witnessing the norm of brands interacting with everyday Twitter users may reinforce a users’ decision to engage with a brand^(26)^. Third, viewing conversations with everyday users that go viral, like clapback tweets, may encourage more people to try to heckle brands^(26)^ in an attempt to engage with the brand. By creating personas, brands are forming an identity that is approachable and offering opportunities to connect on a more intimate level. Brands may, therefore, be fulfilling social needs^(27)^ that provide emotional connectedness and evoke laughter, curiosity and other positive feelings towards brands.

A systematic review of how digital marketing impacts young peoples’ attitudes towards and use of unhealthy products (e.g. ultra-processed food, sugary-sweetened beverages, alcohol and tobacco products) found that interactive marketing endorsed by peers (via social media ‘likes’, comments and reposting) may more strongly incline young people towards these unhealthy products than paid or owned media such as banner ads and websites^(28–31)^. The Federal Trade Commission (FTC) neither requires nor encourages companies to use disclosures (e.g. #ad, #sponsored) on their own social media posts^(32)^, suggesting an urgent need to understand how companies may be leveraging these interactive platforms to engage with consumers in ways that are potentially more powerful than television advertising. This study begins to address these gaps by: (1) identifying the prevalence of personification strategies used in tweets by ten food and beverage brands, (2) quantifying the number of retweets, ‘likes’ and comments given to personality and control tweets and (3) comparing advertising spending data and responses to the retweets per follower ratio. To our knowledge, this is the first study to quantify personification strategies used by food and beverage companies on Twitter and compare user responses to those personality tweets.

## Methods

**Fig. 1:**
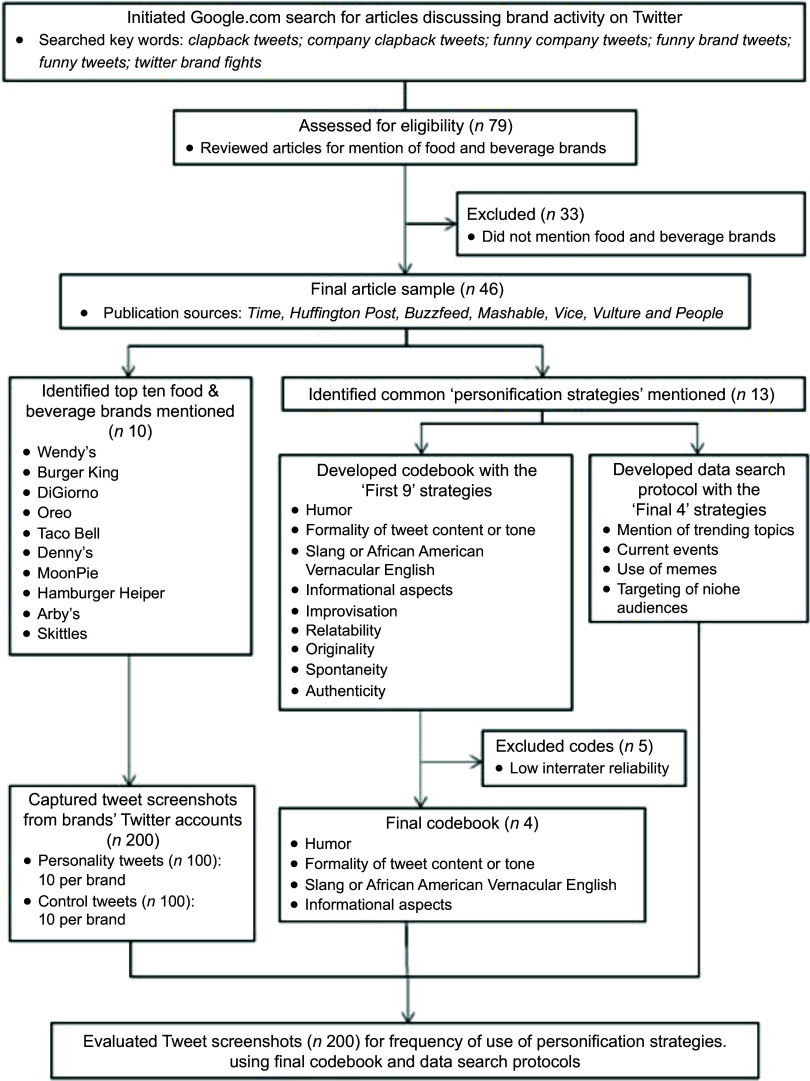
Flow chart describing the identification of tweets for analysis and data collection

To develop a codebook that would enable us to identify and analyse tweets that exemplified a personality (*n* 100) and control tweets (*n* 100), we first conducted a Google search using the following key terms: *twitter brand fights, funny company tweets, funny brand tweets, funny tweets, clapback tweets* and *company clapback tweets*. The resulting articles^(13,15,33–39)^ provided us with our final sample of food and beverage brand Twitter accounts as well as key terms (i.e. codebook codes) that describe the elements of a ‘personality tweet’. Our online search generated a list of 79 media articles. Thirty-three articles were not related to food/beverage brands. The forty-six remaining articles that mentioned food and beverage brands were published by sources like *Time, Forbes*, *Buzzfeed, Mashable, Vice, Vulture* and *People*^(13,15,33–39)^. See Figure 1 for a flow chart of the full data collection process.

The authors developed the codebook by following methods to conduct valid content analyses suggested by Lombard and colleagues^(40)^. Codebook items assessed the presence of key terms and themes from the media articles: (1) humour (e.g. ‘let’s declare war on the sun’ tweet by Moonpie), (2) formality of tweet content (e.g. ‘Try our delicious biscuits’.), (3) slang or African American Vernacular English (e.g. ‘y’all on fleek’), (4) product centring (e.g. ‘The Baja Freeze is back!’), (5) improvisation in response to another brand (e.g. In response to ChickfilA: ‘Bun + Chicken + Pickles = all <3 for the original’, Popeyes: ‘… y’all good?’), (6) relatability (e.g. ‘Who else struggles this hard? #adulting’), (7) originality (e.g. ‘For the 3^rd^ time this month my idea for ‘Moonpie in a can’ was unanimously voted down by everyone in the company’), (8) spontaneity (e.g. ‘it’s a sign of greatness to go by one name: Cher, Oprah, Waldo, Denny’s’) and (9) authenticity (e.g. ‘when you’re roommate eats all your helper without asking’ (pic of the Hamburger Helper glove tearing a heart in two in the style of the Kanye West *808s and Heartbreak* album cover)). Three research assistants completed pilot coding for 10·0 % of the tweets to establish interrater reliability. Discrepancies were resolved by discussing the differences and coming to a consensus, and then those three coders proceeded to code the remainder of the sample. We used Krippendorf’s alpha to assess the intercoder reliability^(40)^.

The final sample included ten brands that were mentioned in the articles: Wendy’s (@Wendys), Burger King (@BurgerKing), DiGiorno (@DiGiorno), Oreo (@Oreo), Taco Bell (@TacoBell), Denny’s (@Dennys), MoonPie (@Moonpie), Hamburger Helper (@Helper), Arby’s (@Arbys) and Skittles (@Skittles).

### Selecting tweets for qualitative analyses

After selecting the sample of ten food and beverage brand Twitter accounts, one author (TG) captured a total of 200 screenshots of tweets that were posted between September 2016 and August 2019. TG used the keywords in the codebook to capture ten screenshots of personality tweets per brand, and the senior author (MB) reviewed that the personality tweets reflected the themes of the codebook. After identifying those 100 personality tweets, TG identified 100 control tweets. The longest time period between when a brand posted a personality tweet and control tweet in our sample was 24 h.

This search process yielded the final sample of 200 screenshots of tweets. The difference between the personality tweets and control tweets is that personality tweets utilise sarcastic or dry humour, heckling, or other types of personification, and the product is either not featured or not the central focus of the post (online supplementary material, Supplementary Figure 2). In contrast, the control tweets focus primarily on showcasing the product and communicating information about the product itself or traditional promotion techniques (e.g. scholarship contests, free meals for veterans), and references to general, widely known events (e.g. New Years, start of spring). See online supplementary material, Supplementary Figures 3 and 4 for examples of control tweets.

Collection of the tweets occurred at least 12 h after the account posted the tweet to ensure that tweets that had not been a part of the previous day’s prime time for engagement were not included. Two consulting and analytics companies reported that the half-life of a tweet is about 20 min, meaning that after only an hour, a tweet is approaching its maximum engagement^(41,42)^. While we could not find more reputable sources about the life of a tweet, the American Marketing Association reported that 8 am to 4 pm on weekdays yielded the most consistent engagement for posting to social media^(43)^. We excluded the following tweets from analysis: (1) tweets by companies that appeared in comments or replies and not on the main company twitter page; (2) tweets pinned at the top on the twitter page and (3) tweets that were posted less than 12 h before the screenshot was taken and therefore did not have enough time to generate a response from followers.

### Collecting descriptive data

We also collected objective data to confirm interactions with everyday Twitter users, use of trending topics, current events, memes and the targeting of niche audiences (online supplementary material, Supplementary Figure 1), as well as the real-time quality of responses by the companies to tweets from followers or other brands. To confirm interactions with everyday Twitter users, we searched the twitter handle of the account tagged in the tweet and confirmed their account had no verification badge, indicating the user was not a celebrity, another food or beverage company, or any other company that might interact with these brands.

We also searched www.Google.com to confirm when companies were using trending topics (e.g. #BestMovieLineEver), current events, memes (e.g. creating your own Daenerys Targaryen title from Game of Thrones) and the targeting of niche audiences (e.g. marching band fans and members). We also searched the site www.knowyourmeme.com to confirm the presence or absence of memes.

### Recording likes, comments and retweets

To evaluate the popularity of tweets, we recorded the number of ‘likes’, comments and retweets of each tweet at the time of data collection. To better understand the influence of personality on the *spread* of tweets, we also created a ratio of the number of retweets per follower (i.e. ‘number of retweets’ divided by ‘number of followers’).

We chose to examine retweets per follower instead of the absolute number of retweets to account for the overall exposure of a brand’s tweets. To our knowledge, this measure has not been used in content analyses of Twitter data, but it has been used in network analysis investigations as a stand-alone measure^(44)^ and as part of more complex algorithms^(45)^. Other studies have examined the validity of retweets and followers separately, citing number of retweets or average number of retweets as the most effective predictor of widespread influence on Twitter^(46,47)^.

### Evaluating the impact of advertising spending

To assess the relationship between advertising spending and retweets per follower, we used the Rudd Center Targeted Marketing, Fast Food and Snack F.A.C.T.S. reports to find data on advertising expenditures for Wendy’s, Oreo, Taco Bell, Burger King and Arby’s. The Rudd Center F.A.C.T.S. reports did not include specific advertising spending information on MoonPie or Denny’s, but other sources indicated Denny’s advertising expenditures were $14·3 million^(48)^. We searched reputable sources on marketing and consumer goods (e.g. AdAge, Ad Weekly) and could not access reliable advertising expenditure data for Moonpie, DiGiorno, Hamburger Helper or Skittles. We excluded those four brands from any analysis concerning the impact of advertising spending.

## Results

### Engagement with brands’ tweets

Table 2 depicts the top three tweets from each brand ranked by number of retweets. The top ten most-retweeted tweets in Table 2 are also the top ten most-retweeted tweets overall.

**Table 2:** Personification techniques used in top three tweets from each brand, ranked by the number of retweets

Company	Number of retweets	Number of likes	Number of comments	Product-related personification*	Use of humour	Non-mainstream group reference	Trending topic reference	Informal or trendy language
Wendy’s	40 000	213 000	3700			–	–	
Wendy’s	26 000	99 000	2200	–				
Wendy’s	23 000	104 000	1100			–	–	–
Denny’s	22 000	86 000	1300		–			–
Hamburger Helper	16 000	15 000	244				–	
Moonpie	7300	74 000	285	–				–
Moonpie	3700	17 000	284	–				–
Moonpie	2600	28 000	183			–	–	–
Taco Bell	2000	6400	310	–	–			–
Denny’s	1300	8900	691			–	–	–
Arby’s	1300	7800	312		–		–	
Arby’s	1000	4800	52				–	–
Arby’s	901	6900	194			–	–	
Hamburger Helper	737	2800	40			–	–	–
Oreo	702	4000	2200		–			
Hamburger Helper	656	2400	29				–	–
Taco Bell	595	6600	101		–	–		–
Burger King	550	2200	992		–	–	–	
Burger King	518	3600	188			–	–	–
Taco Bell	472	1000	239	–	–			
Burger King	467	2800	137			–	–	–
Denny’s	311	2300	29				–	–
Oreo	247	994	37					
Skittles	112	894	43	–		–		–
DiGiorno	97	914	95	–	–	–	–	
Skittles	89	362	28			–	–	–
DiGiorno	87	952	93					–
Skittles	63	360	59	–		–		–
Oreo	56	118	9		–	–		
DiGiorno	45	787	29			–	–	–
**Totals**	**152 905**	**703 881**	**15 203**					
Mean				**22**	**21**	**14**	**13**	**11**
sd				**73·3**	**70·0**	**46·7**	**43·3**	**36·7**

* This column refers to the personification strategy described as ‘product centering’ in the text. This specific column refers to the occurrence of a tweet that primarily existed to personify the brand but still mentioned or pictured the product, as opposed to primarily existing to promote the product, or not mentioning the product at all.

Personality posts were retweeted 123 013 times, ‘liked’ 732 076 times and commented on 14 806 times. Control posts were retweeted 61 044 times, liked 256 105 times and commented on 14 572 times.

Hamburger Helper had the highest number of retweets per follower across all 200 tweets (0·630), meaning that 63 % of all followers retweeted the posts. This was followed by MoonPie (0·078) and Denny’s (0·050).

Personality tweets had higher retweets per follower (0·0118) than control tweets (0·0059) across the 100 tweets from the 10 brands. This means that 1·18 % of followers across all ten brands retweeted the personality posts and 0·59 % of all followers of these brands retweeted the control tweets.

### Thematic content of brands’ tweets

Table 3 evaluates the thematic content of personality strategies used in personality tweets compared to control tweets. For the thematic content evaluation, we used Krippendorf’s alpha to assess the intercoder reliability. Two codebook items – informal/trendy language (collapsed from the items ‘formality of tweet content’ and ‘slang or African American Vernacular English’) and humour – had a value of 0·5. The codebook item pertaining to informational aspects had a Krippendorf’s alpha value of 0·7. The variables assessing improvisation, relatability, originality, spontaneity and authenticity were excluded due to lower reliability.

**Table 3:** Thematic content evaluation of personification strategies comparing personality and control tweets, ranked by retweets per follower

Brand	Retweets per follower	Followers (*n*)	Humour				Informal or trendy language				Personification and product mention*			
			Personality tweets		Control tweets		Personality tweets		Control tweets		Personality tweets		Control tweets	
			*n*	%	*n*	%	*n*	%	*n*	%	*n*	%	*n*	%
Hamburger Helper	0·63031	28 900	10	100	5	50	10	100	10	100	10	100	8	80
MoonPie	0·07836	301 300	10	100	9	90	10	100	10	100	7	70	5	50
Denny’s	0·05025	506 000	9	90	6	60	8	80	4	40	8	80	6	60
Wendy’s	0·02751	3 600 000	9	90	2	20	8	80	4	40	8	80	0	0
Arby’s	0·00639	854 500	6	60	7	70	7	70	2	20	10	100	3	30
DiGiorno	0·00369	150 300	10	100	5	50	10	100	1	10	9	90	1	10
Taco Bell	0·00270	1 900 000	3	30	2	20	7	70	2	20	7	70	1	10
Burger King	0·00249	1 800 000	10	100	3	30	10	100	3	30	8	80	1	10
Oreo	0·00187	873 900	5	50	2	20	1	10	1	10	9	90	8	80
Skittles	0·00125	403 000	9	90	8	80	9	90	6	60	6	60	6	60
Total	0·01767	10,417,900	81	81	49	49	80	80	43	43	82	82	39	39
Totals across personality and control tweets *n*(%)			130 (65·0)				123 (61·5)				121 (60·5)			

* This column refers to the personification strategy described as ‘product centering’ in the text. This specific column refers to the occurrence of a tweet that primarily existed to personify the brand but still mentioned or pictured the product, as opposed to primarily existing to promote the product, or not mentioning the product at all.

#### Personality tweets

For the 100 personality tweets, humour was the most popular personification strategy (*n* 81), followed by informal/trendy language (e.g. ‘brb i’m bout to get lost in this sauce;’ *n* 80), trending topics (‘What’s a Skittles gotta do to get their Broadway Musical turned into a movie? #CatsMovie #SkittlesMovie;’ *n* 47), meme posts (*n* 31), interactions with everyday Twitter users (*n* 20) and non-mainstream group references (‘How do you do, fellow planeswalkers’ referencing the obscure ‘Magic the Gathering’ card game; *n* 6).

#### Control tweets

We observed a different pattern among the 100 control tweets, which focused more heavily on promotion of the actual food product than the personality tweets (see Tables 3 and 4). More than half (*n* 52) of tweets, for example, were purely promotions of the food product (i.e. ‘Have you tried the $5 biggie bag? This deal is too good to pass up!’). Control tweets also used some personification strategies, but these tweets were primarily focused on portraying the food product itself. For the control tweets, humour that targeted a wide audience was the most popular strategy (*n* 49), followed by informal/trendy language (*n* 43), trending topics (*n* 39), non-mainstream group references (*n* 9), meme posts (*n* 3) and interactions with everyday twitter users (*n* 1).

**Table 4:** Descriptive data on personification techniques comparing personality and control tweets, ranked by retweets per follower

Brand	Retweets per follower	Followers (*n*)	Trending topic posts				Meme posts				Interactions with everyday Twitter users			
			Personality tweets		Control tweets		Personality tweets		Control tweets		Personality tweets		Control tweets	
			*n*	%	*n*	%	*n*	%	*n*	%	*n*	%	*n*	%
Hamburger Helper	0·63031	28 900	1	10	0	0	3	30	0	0	0	0	0	0
MoonPie	0·07836	301 300	7	70	7	70	3	30	0	0	0	0	0	0
Denny’s	0·05025	506 000	0	0	2	20	0	0	0	0	0	0	0	0
Wendy’s	0·02751	3 600 000	8	80	10	100	5	50	0	0	6	60	0	0
Arby’s	0·00639	854 500	4	40	3	30	1	10	1	10	1	10	0	0
DiGiorno	0·00369	150 300	5	50	0	0	4	40	0	0	2	20	0	0
Taco Bell	0·00270	1 900 000	4	40	4	40	1	10	0	0	7	70	0	0
Burger King	0·00249	1 800 000	1	10	1	10	8	80	0	0	2	20	0	0
Oreo	0·00187	873 900	9	90	10	100	3	30	1	10	2	20	1	10
Skittles	0·00125	403 000	8	80	2	20	3	30	1	10	0	0	0	0
Total	0·01767	10 417 900	47	47	39	39	31	31	3	3	20	20	1	1
Totals across personality and control tweets, *n* (%)			86 (43·0)				34 (17·0)				21 (10·5)			

#### Descriptive statistics of personality and control tweets

Table 4 compares the descriptive data on personification techniques used in personality tweets compared to control tweets. For the three most-retweeted tweets per brands (*n* 30), eighteen of those were personality tweets (e.g. a clapback in response to a tweet by Popeyes ‘I guess that means the food’s as dry as the jokes’) and twelve were control tweets (see Table 2), usually with massive giveaways (e.g. ‘The Warriors stole game 2 in the NBA finals, which means free Tacos for everyone 18^th^!’). Across all thirty of the most-retweeted posts, humour was the most popular personification strategy (70 %; *n* 21), followed by non-mainstream group references (46·7 %; *n* 14). (Percentages exceed 100 because 1 tweet might demonstrate multiple personification strategies at once).

### Impact of brand advertising spending

**Fig. 2:**
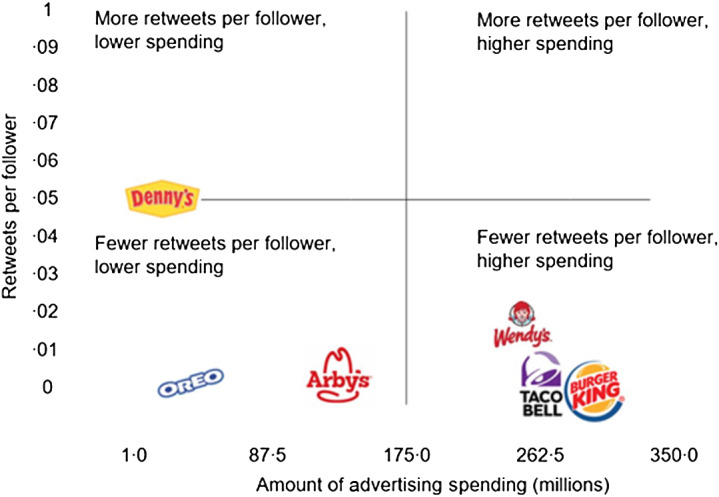
Relationship between advertising spending and retweets per follower

Figure 2 depicts the relationship between advertising spending and retweets per follower, suggesting higher advertising expenditures do not always correlate with a higher number of retweets per follower.

## Discussion

Food brands used a diverse portfolio of advertising strategies on Twitter – including human personification techniques – to interact with consumers with humour, informal/trendy language and trending topics. When we compared responses between personality tweets and control tweets, findings revealed substantial differences between the number of retweets, likes and comments. Personality tweets, for example, garnered double the retweets and nearly triple the number of ‘likes’ compared to control tweets. This new form of advertising is a public health concern because it may blur the line between entertainment and advertising^(49)^, thereby increasing exposure to unhealthy food and beverage ads that increase risk for poor diet. Because these tweets are coming from the brands’ accounts, they are not required to use the ‘Ad’ designation as required by the FTC for other entities which could enable ads to slip under the radar of people’s ability to recognise the promotional nature^(50,51)^.

Companies in our sample that spent the most on advertising had similar ratios of retweets per follower than the majority of those that had lower advertising spending, suggesting high advertising budgets do not necessarily correlate with more retweets per followers. Moreover, all brands in our sample were popular on Twitter, even though some – like Hamburger Helper, Denny’s and MoonPie – are considered less popular among consumers. The media articles we found that described the ‘brand personality’ phenomenon mentioned that obscure brands often have the strongest personalities (i.e. obscure brands like Moonpie might not have as many followers as a larger brand like Skittles, but Moonpie’s tweets may be so engaging that they spread rapidly through retweets).

Whereas prior studies of brand personality on social media have noted differences in the success of adopting broad personality traits on Twitter and Instagram^(18,26,52)^, our study is the first to capture the nuances of brand personification and use personification to examine spread of tweets. In a content analysis of brand-related Twitter posts, researchers examined how brand personalities were perceived on and off of social media and found that brands that presented as more ‘rugged’ received more retweets than brands that presented primarily with different personality traits, and brands that presented as more sincere received less retweets than brands that presented primarily with different personality traits^(18)^. But that study used broad search terms, limiting their ability to capture the intricacies of anthropomorphic advertising. Companies in our sample, for example, harnessed the nimble and flexible tools of social media to incorporate trending memes, slang, pop culture topics and target niche audiences. Another study investigated the number of times children and adolescents viewed food and beverage advertising, while using two social media apps for 5 min each and estimated that children and adolescents are exposed to food marketing on social media an average of 20 and 189 times per week, respectively^(53)^.

This study has some limitations. First, we included only ten brands in our analysis and only twenty tweets from each of the brands. While these brands were determined to be most representative of a trend, it does exclude other brands that may use a variety of methods to gain retweets and followers with similar results. Second, we adjusted available ad spending data for inflation for two brands in order to estimate standardised total expenditures in 2017. This means our ad spending estimates for some brands may differ slightly from their actual spending data. Additionally, improving content analysis methods to capture both the discussed nuances in measures we included in our analysis and those that were excluded due to low reliability (e.g. authenticity, spontaneity, etc.) is another important step. These concepts were difficult to operationalise, and the development of better definitions and clearer examples could improve this in the future.

Future studies should determine the number of Twitter users who do not follow food and beverage brands but are exposed to this advertising through retweets to reveal the reach of these strategies. Experimental research should also examine the extent to which personality tweets increase engagement and product purchases among young adults and younger audiences. Given these brands can personify themselves in ways that may fulfill some of their followers’ social needs for connection, experimental research should examine the extent to which brand loyalty and purchases increase as a result of interactions between consumers and brands that cultivate an interactive persona.

In sum, brands that embody distinct personalities on Twitter are uniquely powerful because they prompt millions of followers to retweet content to peers, some of whom may not be following the brands. Because Twitter enables brands to reach a global audience, this form of promotion may increase international visibility of energy-dense, nutrient-poor products that are promoted primarily by US-based companies. Although more than a dozen countries have policies that limit child-targeted food advertising, the vast majority of those countries do not address online food marketing^(54)^. Personality tweets appear to appeal to consumers more than other tweets, which is concerning if this form of promotion increases the purchases and consumption of food and beverages that contribute to poor diet and diet-related diseases. This content analysis of ten brands is the first step in understanding how this phenomenon influences consumers’ purchases of unhealthy foods and beverages. More research is needed to explore the strategies these brands use and their impact as their departure from recognisable traditional advertising messaging makes it particularly ripe for negative impacts on population health, especially with young adult Twitter users.

## Supplementary material

For supplementary material accompanying this paper visit https://doi.org/10.1017/S1368980021001439. click here to view supplementary material
