# Supplementary material for: Brands with personalities – good for businesses, but bad for public health? A content analysis of how food and beverage brands personify themselves on Twitter
Source: Public Health Nutr. 2021 Apr 6;25(1):51–60. doi: 10.1017/S1368980021001439 (PMC8825980; doi:10.1017/S1368980021001439)

Supplementary Figure 1. Examples of Arby’s Personality Tweet Demonstrating Targeting of Niche Audiences


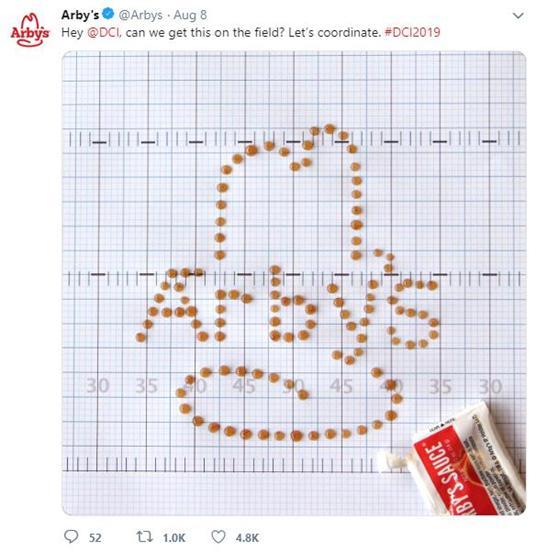

Supplement: Supplementary file 1 [file S1368980021001439sup.zip › S1368980021001439sup001.docx]
