# Supplementary material for: Brands with personalities – good for businesses, but bad for public health? A content analysis of how food and beverage brands personify themselves on Twitter
Source: Public Health Nutr. 2021 Apr 6;25(1):51–60. doi: 10.1017/S1368980021001439 (PMC8825980; doi:10.1017/S1368980021001439)
Supplement: Supplementary file 1 [file S1368980021001439sup.zip › S1368980021001439sup003.docx]

| **Supplementary Table 1.** Characteristics of those with qualified and unqualified urine samples | | | |
| --- | --- | --- | --- |
|  | Unqualified (N=177) | Qualified (N=561) | t, Z, X^2^/P |
| Age (Mean±SD) | 49.1±9.5 | 52.7±9.3 | 4.428/<0.001 |
| 35-44 (n, %) | 51 (28.8) | 102 (18.2) | 11.466/0.003 |
| 45-59 (n, %) | 102 (57.6) | 341 (60.8) |  |
| ≥60 (n, %) | 24 (13.6) | 118 (21.0) |  |
| Gender (men, %) | 91 (51.4) | 239 (42.6) | 4.224/0.040 |
| Education (n, %) |  |  | 0.877/0.645 |
| Primary and lower | 66 (37.5) | 213 (38.0) |  |
| Junior | 52 (29.5) | 147 (26.2) |  |
| Senior and higher | 58 (33.0) | 201 (35.8) |  |
| Cigarette consumption (n, %) | 46 (26.1) | 101 (18.2) | 5.241/0.022 |
| Alcohol intake (n, %) | 39 (22.2) | 137 (24.6) | 0.451/0.502 |
| Body mass index category (n, %) |  |  | 12.848/0.002 |
| ＜25kg/m² | 52 (29.4) | 186 (33.2) |  |
| 25-30kg/m² | 62 (35.0) | 250 (44.6) |  |
| ≥30kg/m² | 63 (35.6) | 125 (22.3) |  |
| Abdominal obesity (n, %) | 72 (40.7) | 218 (38.9) | 0.172/0.678 |
| Prevalence of hypertension (n, %) | 87 (49.2) | 278 (49.6) | 0.009/0.926 |
| Prevalence of dyslipidemia (n, %) | 52 (30.4) | 204 (36.8) | 2.353/0.125 |
| Prevalence of diabetes (n, %) | 15 (8.6) | 64 (11.5) | 1.164/0.281 |
| Prevalence of stroke (n, %) | 16 (9.6) | 48 (8.9) | 0.074/0.785 |
| Anti-hypertensive agents (n,%) | 59 (67.8) | 144 (51.8) | 6.887/0.009 |
| Prevalence of MCI (n, %) | 36 (20.7) | 107 (19.1) | 0.221/0.638 |
| Salt intake <6g (n, %) | 101 (64.7) | 137 (24.4) | 89.495/<0.001 |
| Potassium intake ≥3.5g (n, %) | / | 2 (0.4) | 1.000 |
| Sodium to potassium ratio (n, %) |  |  | 0.651/1.00 |
| <1.0 | / | 1 (0.2) |  |
| =1.0 | / | 2 (0.4) |  |
| >1.0 | 156 (100.0) | 558 (99.5) |  |
| Body mass index (Median, Q1~Q3) | 27.8 (24.2, 31.1) | 26.7 (24.1, 29.6) | 2.997/0.003 |
| Systolic Blood Pressure (Median, Q1~Q3) | 129.3 (119.3, 145.5) | 129.7 (118.3, 143.7) | 0.539/0.590 |
| Diastolic Blood Pressure (Median, Q1~Q3) | 83.7 (75.3, 91.3) | 82.3 (74.7, 90.0) | 1.055/0.292 |
| Total MMSE score (Median, Q1~Q3) | 26 (22, 28) | 26 (22, 29) | 0.967/0.333 |
| Serum creatinine (Median, Q1~Q3) | 65.6 (52.7, 82.6) | 71.1 (57.0, 90.0) | 2.120/0.034 |
| Estimated salt intake (Median, Q1~Q3) | 4.8 (3.1, 7.2) | 8.6 (6.1, 11.6) | 10.227/<0.001 |
| Estimated sodium intake (Median, Q1~Q3) | 1.9 (1.2, 2.8) | 3.4 (2.4, 4.6) | 10.227/<0.001 |
| Estimated potassium intake (Median, Q1~Q3) | 0.6 (0.4, 0.8) | 1.2 (0.9, 1.5) | 14.002/<0.001 |
| Individuals unqualified are those with unqualified urine sample (n=175) and incompleteness on MMSE (n=2). | | | |

| **Supplementary Table 2.** Characteristics of subjects after propensity score matching (N=352) | | | |
| --- | --- | --- | --- |
|  | 24-h UNa/K ≤4.80 | 24-h UNa/K >4.80 | Z, X^2^/P |
| Age (Median, Q1~Q3) | 52.4 (46.7, 58.3) | 53.3 (46.0, 59.4) | 0.190/0.849 |
| Gender (men, %) | 65 (36.9) | 72 (40.9) | 0.586/0.444 |
| Education (n, %) |  |  | 0.922/0.631 |
| Primary and lower | 65 (36.9) | 67 (38.1) |  |
| Junior | 43 (24.4) | 49 (27.8) |  |
| Senior and higher | 68 (38.6) | 60 (34.1) |  |
| Occupation (n, %) |  |  | 0.534/0.766 |
| Mental labour | 51 (29.0) | 45 (25.6) |  |
| Physical labour | 110 (62.5) | 116 (65.9) |  |
| Unemployed/unclassified | 15 (8.5) | 15 (8.5) |  |
| Cigarette consumption (n, %) | 28 (15.9) | 34 (19.3) | 0.705/0.401 |
| Alcohol intake (n, %) | 40 (22.7) | 43 (24.4) | 0.142/0.706 |
| Body mass index (Median, Q1~Q3) | 26.3 (23.8, 29.6) | 26.7 (24.5, 29.6) | 0.957/0.339 |
| Estimated glomerular filtration rate (Median, Q1~Q3) | 94.2 (68.0, 104.6) | 88.6 (71.6, 103.8) | 0.072/0.942 |
| Hypertension (n, %) | 84 (47.7) | 88 (50.0) | 0.182/0.670 |
| Dyslipidemia (n, %) | 65 (36.9) | 68 (38.6) | 0.109/0.742 |
| Diabetes (n, %) | 18 (10.2) | 23 (13.1) | 0.690/0.406 |
| Stroke (n, %) | 14 (8.0) | 11 (6.3) | 0.388/0.534 |
| Matched for age, gender, education status, occupation, smoking status, alcohol assumption, body mass index, estimated glomerular filtration rate, hypertension, stroke, dyslipidemia, and diabetes mellitus. | | | |

| **Supplementary Table 3.** MMSE score and prevalence of MCI in subjects by the median of 24-hour urine sodium to potassium ratio after propensity score matching (N=352). | | | |
| --- | --- | --- | --- |
|  | 24-h UNa/K ≤4.80 | 24-h UNa/K >4.80 | Z, X^2^/P |
| Total MMSE score | 27.0 (23.0, 29.0) | 25.0 (21.0, 28.0) | 2.594/0.009 |
| Orientation | 10.0 (9.0, 10.0) | 10.0 (8.0, 10.0) | 1.889/0.059 |
| Registration | 3.0 (3.0, 3.0) | 3.0 (3.0, 3.0) | 1.674/0.094 |
| Attention and Calculation | 4.0 (2.0, 5.0) | 4.0 (1.0, 5.0) | 2.158/0.031 |
| Recall | 3.0 (1.0, 3.0) | 2.0 (1.0, 3.0) | 2.349/0.019 |
| Language and Praxis | 9.0 (7.0, 9.0) | 9.0 (7.0, 9.0) | 1.890/0.059 |
| Prevalence of MCI (n, %) | 22 (12.5) | 45 (25.6) | 9.752/0.002 |
| Matched for age, gender, education status, occupation, smoking status, alcohol assumption, body mass index, estimated glomerular filtration rate, hypertension, stroke, dyslipidemia, and diabetes mellitus. | | | |
